# Microfluidic Isolation of Neuronal-Enriched Extracellular Vesicles Shows Distinct and Common Neurological Proteins in Long COVID, HIV Infection and Alzheimer’s Disease

**DOI:** 10.3390/ijms25073830

**Published:** 2024-03-29

**Authors:** Lynn Pulliam, Bing Sun, Erin McCafferty, Steven A. Soper, Malgorzata A. Witek, Mengjia Hu, Judith M. Ford, Sarah Song, Dimitrios Kapogiannis, Marshall J. Glesby, Daniel Merenstein, Phyllis C. Tien, Heather Freasier, Audrey French, Heather McKay, Monica M. Diaz, Igho Ofotokun, Jordan E. Lake, Joseph B. Margolick, Eun-Young Kim, Steven R. Levine, Margaret A. Fischl, Wei Li, Jeremy Martinson, Norina Tang

**Affiliations:** 1 Department of Laboratory Medicine, University of California, San Francisco, CA 94143, USA; 2 Department of Laboratory Medicine, San Francisco VA Health Care System, San Francisco, CA 94121, USA; sbbmu@hotmail.com (B.S.); erin.m.mccafferty@gmail.com (E.M.); norina.tang@va.gov (N.T.); 3 Department of Chemistry, The University of Kansas, Lawrence, KS 66045, USA; ssoper@ku.edu (S.A.S.); mwitek@ku.edu (M.A.W.); 4 Center of BioModular Multiscale Systems for Precision Medicine, The University of Kansas, Lawrence, KS 66045, USA; 5 Cancer Biology, The University of Kansas Medical Center, Kansas City, KS 66103, USA; m367h922@ku.edu; 6 Bioengineering Program, The University of Kansas, Lawrence, KS 66045, USA; 7 Department of Mental Health, San Francisco VA Health Care System, San Francisco, CA 94121, USA; judith.ford@ucsf.edu (J.M.F.); sarahsong137@berkeley.edu (S.S.); 8 Department of Psychiatry and Behavioral Sciences, University of California San Francisco, San Francisco, CA 94143, USA; 9 Laboratory of Clinical Investigation, Intramural Research Program, National Institute on Aging, National Institutes of Health, Baltimore, MD 20892, USA; kapogiannisd@mail.nih.gov; 10 Department of Medicine, Weill Cornell Medical College, New York City, NY 10021, USA; mag2005@med.cornell.edu; 11 Department of Family Medicine, Georgetown University School of Medicine, Washington, DC 20007, USA; djm23@georgetown.edu; 12 Department of Medicine, University of California San Francisco, San Francisco, CA 94143, USA; phyllis.tien@ucsf.edu (P.C.T.); heather.freasier@ucsf.edu (H.F.); 13 Department of Medicine, San Francisco VA Health Care System, San Francisco, CA 94121, USA; 14 Department of Medicine, Cook County Health, Chicago, IL 60612, USA; afrench@cookcountyhhs.org; 15 Department of Epidemiology, Johns Hopkins Bloomberg School of Public Health, Baltimore, MD 21205, USA; hmckay4@jhu.edu; 16 Department of Neurology, University of North Carolina at Chapel Hill School of Medicine, Chapel Hill, NC 27599, USA; monica.diaz@neurology.unc.edu; 17 Department of Medicine, Emory University School of Medicine, Atlanta, GA 30322, USA; iofotok@emory.edu; 18 Department of Internal Medicine, University of Texas Health Science Center at Houston, Houston, TX 77030, USA; jordan.e.lake@uth.tmc.edu; 19 Department of Molecular Microbiology and Immunology, Johns Hopkins Bloomberg School of Public Health, Baltimore, MD 21205, USA; jmargol1@jhu.edu; 20 Department of Medicine, Northwestern University Feinberg School of Medicine, Chicago, IL 60611, USA; e-kim@northwestern.edu; 21 Department of Neurology, State University of New York College of Medicine and Downstate Medical Sciences University, Brooklyn, NY 11203, USA; steven.levine@downstate.edu; 22 Department of Medicine, University of Miami, Miami, FL 33136, USA; mfischl@med.miami.edu; 23 Department of Clinical and Diagnostic Sciences, University of Alabama, Birmingham, AL 35294, USA; wli@uab.edu; 24 Department of Infectious Diseases and Microbiology, Graduate School of Public Health, University of Pittsburgh, Pittsburgh, PA 15260, USA; jmartins@pitt.edu

**Keywords:** L1CAM, extracellular vesicles, OLINK, long COVID, HIV, Alzheimer’s disease, microfluidics

## Abstract

Long COVID (LongC) is associated with a myriad of symptoms including cognitive impairment. We reported at the beginning of the COVID-19 pandemic that neuronal-enriched or L1CAM+ extracellular vesicles (nEVs) from people with LongC contained proteins associated with Alzheimer’s disease (AD). Since that time, a subset of people with prior COVID infection continue to report neurological problems more than three months after infection. Blood markers to better characterize LongC are elusive. To further identify neuronal proteins associated with LongC, we maximized the number of nEVs isolated from plasma by developing a hybrid EV Microfluidic Affinity Purification (EV-MAP) technique. We isolated nEVs from people with LongC and neurological complaints, AD, and HIV infection with mild cognitive impairment. Using the OLINK platform that assesses 384 neurological proteins, we identified 11 significant proteins increased in LongC and 2 decreased (BST1, GGT1). Fourteen proteins were increased in AD and forty proteins associated with HIV cognitive impairment were elevated with one decreased (IVD). One common protein (BST1) was decreased in LongC and increased in HIV. Six proteins (MIF, ENO1, MESD, NUDT5, TNFSF14 and FYB1) were expressed in both LongC and AD and no proteins were common to HIV and AD. This study begins to identify differences and similarities in the neuronal response to LongC versus AD and HIV infection.

## 1. Introduction

Following the early acute phase of the COVID-19 pandemic, there continue to be reports of individuals with SARS-CoV-2 infection suffering lingering sequelae in spite of vaccination, which has been termed long COVID (LongC), also called “post-acute sequelae of SARS-CoV-2 infection” (PASC). Many individuals with LongC complain of neurological symptoms, including “brain fog”, memory loss, anxiety and depression [1,2,3,4,5]. At least 65 million people worldwide have LongC [6]. Cases of LongC in adults over 18 years old in the U.S. vary between 2 and 10% as of 2022 [7]. This was defined as self-reported symptoms over 3 months that were not present before infection. We now know that prolonged memory deficits, concentration difficulties and anxiety associated with COVID-19 infection can last for over 2 years [7,8,9] and have a significant impact on quality of life. A recent review of the literature suggests an increased susceptibility to Alzheimer’s disease (AD) after COVID-19 infection [10]. AD is the most common dementia and affects over 6 million Americans 65 years old and above. Death from AD has increased by 16% during COVID-19 [11]. It is the sixth leading cause of death in the U.S. and payments for healthcare for people over 65 and older with AD or other dementias were estimated at USD 345 billion [11,12]. Cognitive impairment in HIV has changed dramatically with effective antiretroviral therapy and there is ongoing controversy as to whether cognitive impairment exists with controlled viral suppression or whether it is a result of co-morbidities and aging [13]. As people with HIV age, it may be difficult to differentiate HIV-associated cognitive impairment from AD.

Studies to differentiate and compare cognitive impairment in LongC and HIV with AD are just beginning. These are especially timely studies since people with LongC may be at risk for an accelerated brain aging process. To evaluate neuronal health in real time, neuronal-enriched extracellular vesicles (nEVs) were isolated from plasma using antibodies against L1CAM. Reports of elevated neurotoxic proteins in nEVs, such as amyloid beta (Aβ), neurofilament light and tau phosphorylated at threonine-181 (pTau181), have been shown in AD [14,15], traumatic brain injury [16,17,18] and HIV cognitive impairment [19]. We previously reported that nEVs from LongC had some of the same elevated toxic proteins as nEVs from individuals with AD [20]. Recently, neuropathologic findings suggest that SARS-CoV-2 and AD have similar neuroinflammatory pathways [21,22,23]. While HIV cognitive impairment affects a subset of individuals with HIV infection, LongC may also be associated with cognitive impairment in a subset of SARS-CoV-2-infected people that may herald a neurodegenerative process [24]. With the propensity of SARS-CoV-2 and HIV viral infections to exacerbate aging, we sought to compare the nEV proteins from people with these two viral infections with those from people diagnosed with AD.

Processing enough plasma nEVs to perform multiple analyses has been difficult because of the small number in circulation and the abundance of soluble L1CAM (sL1CAM) in the plasma. To address this, we developed a hybrid technique involving an initial precipitation step to isolate total EVs (tEVs) and remove sL1CAM. This is followed by an automated microfluidic platform for enriching nEVs at higher concentrations, which is essential for downstream analyses. Using an Olink proximity extension assay (PEA), we expanded the number of targets to 384 neurologic proteins for comparing the nEVs from the three cohorts. Using this panel, we found no common proteins between HIV and AD, and one shared protein (BST1) between HIV and LongC. AD and LongC had six common proteins elevated in people with cognitive impairment (MIF, ENO1, MESD, NUDT5, TNFSF14 and FYB1).

## 2. Results

### 2.1. Hybrid Microfluidic Affinity Purification (EV-MAP) Characterization

**Figure 1:**
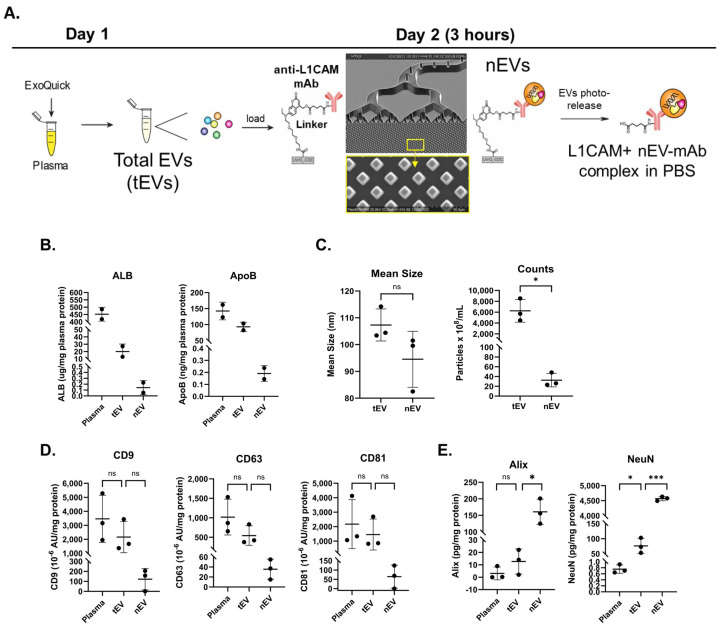
Isolation and characterization of L1CAM+ EVs (nEVs). (**A**) Total EVs were precipitated from plasma using ExoQuick and then loaded onto a microfluidic chip decorated with anti-L1CAM mAbs. Captured nEVs were photoreleased into phosphate-buffered saline. (**B**) Aliquots of unlysed plasma, tEVs and L1CAM+ EVs were assayed by ELISA for albumin and ApoB proteins. *n* = 2. (**C**) EV sizes and concentrations determined by NTA using ZetaView. (**D**) Tetraspanins were quantified using MSD. (**E**) Alix and NeuN were quantified using ELISA with results showing significantly increased protein levels of these exosomal and neuronal markers in nEVs. For panels **C**–**E**, *n* = 3; group differences were compared using paired *t*-tests (two-sided, equal variance). Horizontal bars indicate group means. *p*-value symbols are as follows: ns (*p* > 0.05), * (*p* ≤ 0.01) and *** (*p* ≤ 0.0001).

We set out to streamline the isolation of neuronal-enriched L1CAM+ EVs and to optimize the number of nEVs for multiple analyses. We used a combination of precipitation and microfluidics, which we call “hybrid EV-MAP”, to enrich for nEVs. Hybrid EV-MAP entails the purification of tEVs from plasma using polymer precipitation, and then loading the tEVs onto EV-MAP thermoplastic microfluidic chips [25] which contain a dense array of micropillars that are decorated with monoclonal antibodies to L1CAM (Figure 1A). EV-MAP technology has been used to isolate tumor-specific biomarkers and T cell EVs associated with acute ischemic stroke from plasma or serum [25] By loading tEVs instead of plasma onto the EV-MAP microfluidic chips, co-isolated contaminants, such as albumin and lipoproteins, which are abundant in the plasma, are significantly reduced (Appendix A). In addition, because sL1CAM is abundant in the plasma, by first precipitating the tEVs before loading onto the EV-MAP chips, fewer sL1CAM molecules will bind to the antibodies on the micropillars, thus increasing the capture of L1CAM+ EVs on the micropillars of the chips (Appendix A). After affinity isolation, the nEVs are photo-released from the micropillars into phosphate-buffered saline and further characterized according to the MISEV requirements [26,27]. According to MISEV 2018 guidelines [26], quantification of EVs may be determined by various methods including nanotracking particle analysis (NTA), total protein amount and/or quantification of specific molecules by ELISA, while analysis of single EVs may entail the use of electron microscopy (EM). In addition, protein-content-based EV characterization must include analyses of at least one protein in each one of these categories: (1) transmembrane or GPI-anchored proteins associated with the plasma membrane or endosomes, such as tetraspanins (e.g., CD9, CD63, CD81); (2) cytosolic proteins recovered in EVs, such as Alix; and (3) non-EV co-isolated structures, such as abundant plasma proteins and lipoproteins (e.g., ALB, ApoB).

To evaluate the presence of co-isolated protein contaminants, unlysed plasma, tEVs and isolated L1CAM+ nEVs were subjected to ELISAs for albumin and apolipoprotein B (Figure 1B). Hybrid EV-MAP greatly diminished both proteins in the nEV preps. EVs were further characterized by NTA for size and concentration (Figure 1C). There was no difference in size between tEVs and nEVs. By particle counts, nEVs constituted less than 1% of tEVs. Three typical EV markers, tetraspanins CD9, CD63 and CD81, were detected in the EV preps, with the nEVs showing tetraspanins at roughly 10% of the level seen in the tEVs (Figure 1D). By transmission EM, the appearance of the nEVs was consistent with an exosome size range of 50–150 nm and cup-shaped morphology (Appendix B). Lysed nEVs were analyzed by ELISA for Alix (an exosome marker) and NeuN (a neuronal marker) and both were significantly increased compared to tEVs, demonstrating that these are exosomes and are neuronally enriched (Figure 1E).

### 2.2. Participant Information

The characteristics of the clinical samples are shown in Table 1. There were more males than females in the LongC group. The cohorts were age-matched to their controls. All plasma was collected in EDTA tubes, aliquoted and stored at −80 °C until use. The HIV+ samples were obtained from the MACS/WIHS combined cohort study (MWCCS) [28] and were from people classified as having mild cognitive impairment (MND). The HIV controls (HIV-C) were from HIV+ individuals with normal cognition (NPN), all age- and sex-matched within 4 years. The LongC subjects all had neurological complaints after infection, which included one or more of the following: brain fog, difficulty with concentration, increased anxiety or increased depression. All LongC participants were vaccinated against SARS-CoV-2 except one male. For the LongC control subjects (LongC-C), we used control plasma from participants in the MWCCS who were HIV- with NPN and collected before the pandemic. The LongC and their respective controls were not age- and sex-matched due to sample availability. In the LongC group, there were 12 males and 4 females, while its control group (LongC-C) had 8 males and 8 females. The age range for the LongC group was 26–65 years, and for LongC-C, the range was 46–61 years. The AD cohort and controls were sex- and age-matched within 3 years and were significantly older than both the HIV and LongC participants. The AD cohort consisted of participants selected from NIH IRB-approved clinical studies run at the National Institute of Aging (NIA, Baltimore, MD, USA). Corresponding controls were healthy, cognitively unimpaired individuals participating in NIA clinical trials. Ages between LongC, LongC-C, HIV and HIV-C groups were similar.

### 2.3. Characterization of nEVs

Since soluble L1CAM is abundant in the plasma, we chose to isolate tEVs from plasma. When tEVs were isolated first, followed by microfluidic affinity enrichment using L1CAM, ApoB and albumin were greatly diminished (Appendix A) and nEVs were more abundant.

**Figure 2:**
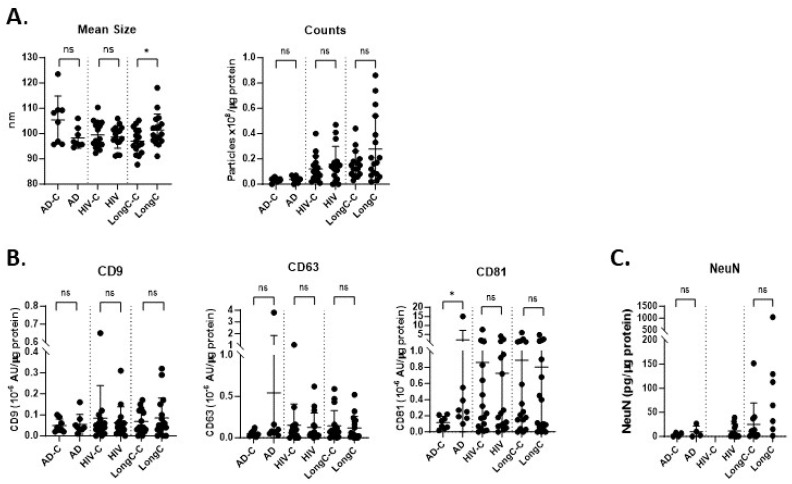
Characterization of nEV samples from HIV, LongC and AD cohorts. (**A**) There were no differences between AD and HIV cohorts when compared with their respective controls for particle size and concentration. The sizes of nEVs in the LongC group were larger than the healthy controls. (**B**) Tetraspanins were quantified using MSD. CD81 was increased on AD nEVs compared to age-matched AD controls: *p* = 0.0104 when all data values were included, and *p* = 0.0205 when the one high outlier was omitted. (**C**) The neuronal marker, NeuN, was present in nEVs. Mann–Whitney tests (unpaired, non-parametric, two-tailed) were used for all comparisons between disease and respective control groups. *p*-value symbols are as follows: ns (*p* > 0.05) and * (*p* ≤ 0.05). For panels **A**,**B**, sample numbers in each group were as follows: AD-C (*n* = 8), AD (*n* = 8); HIV-C is HIV+, NPN (*n* = 16), HIV+ is HIV+, MND (*n* = 16); LongC-C (*n* = 16), LongC (*n* = 16). For (**C**) sample numbers in each group were fewer due to insufficient plasma availability: AD-C (*n* = 4), AD (*n* = 4); HIV-C is HIV+, NPN (*n* = 0), HIV is HIV+, MND (*n* = 11); LongC-C (*n* = 11), LongC (*n* = 7).

nEVs were isolated from all cohort plasma using the hybrid EV-MAP technique described above (Figure 1A). NTA results showed that the size of the particles derived from the LongC samples was larger than their controls. There were no differences in particle concentrations between the cohorts (Figure 2A). We quantified three tetraspanins that are associated with EVs using a multiplex immunoassay from Meso Scale Discovery (MSD). Only CD81 was elevated in nEVs from individuals with AD (Figure 2B: *p* = 0.01 when all data values were included, and *p* = 0.02 when the one high outlier was omitted). The neuronal marker NeuN was not significantly different between the groups but trending higher for the LongC group (Figure 2C, *p* = 0.069). HIV-negative controls were not performed for NeuN as there was insufficient plasma. These results indicate that the material purified using the hybrid EV-MAP technique contained neuronal-enriched EVs.

### 2.4. Proximity Extension Assay (PEA) Results

Protein concentrations of nEVs were determined and samples were adjusted to 0.5 mg/mL with PBS. A PEA panel with 384 neurological proteins from Olink was run. Each group was analyzed separately with its controls for differentially expressed (DE) proteins (Table S1). There were 362 targets that passed the Olink quality check criteria. Only 145 proteins were detected in at least 50% of the samples and, thus, were included in the analyses.

**Figure 3:**
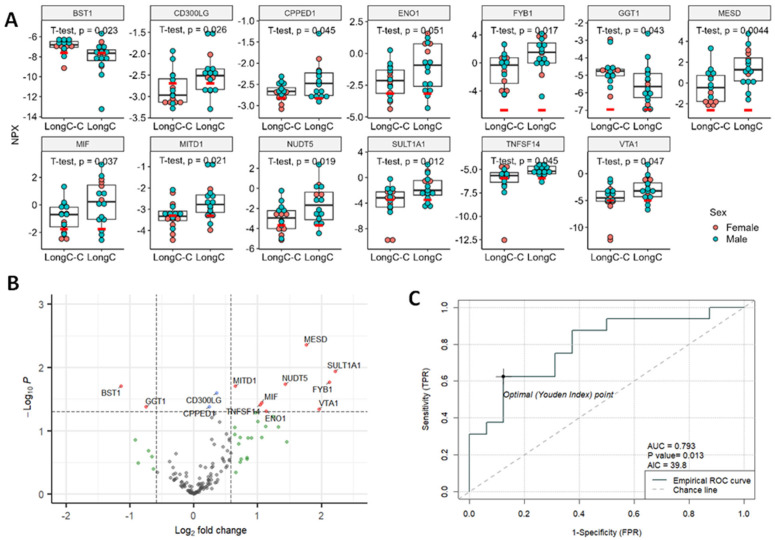
Significant DE proteins for LongC. (**A**) Dot plots of DE proteins. Red bars indicate the levels of lower limit of detection (LOD) of the target. Unpaired, two-sided, unequal variance *t*-tests were used for comparisons of controls to LongC. (**B**) Volcano plot. Vertical dashed lines indicate fold change of 1.5; horizontal line indicates *p* = 0.05. Red dots indicate proteins with fold changes >1.5 or <−1.5, and *p* values < 0.05; blue dots indicate proteins with fold changes of −1.5 to 1.5 and *p* values < 0.05; green dots indicate fold changes >1.5 or <−1.5, and *p* values ≥ 0.05; and gray dots indicate proteins with fold changes of −1.5 to 1.5, and *p* values ≥ 0.05. Red dots indicate significant DE proteins. Blue, green and gray dots indicate non-DE proteins. (**C**) Receiver-operating characteristic (ROC) curve of the best predictor, MESD. NPX, normalized protein expression; AUC, area under the curve; AIC, Akaike information criterion; TPR, true positive rate, and FPR, false positive rate.

The 13 DE proteins in LongC that showed significant differences from controls are shown in Figure 3A. A volcano plot (Figure 3B) shows 11 DE proteins that were significantly increased, and 2 proteins, BST1 and GGT1, that were decreased. A receiver–operating characteristic (ROC) curve was determined for the potential diagnostic capacity with the best predictor, MESD, which had an area under the curve (AUC) of 0.793 (Figure 3C). The Akaike information criterion (AIC) was used to determine the best model for this small data set. The lower the AIC, the better. The ROC curve information for four additional best predictors for LongC is shown in Table S2. The five best predictors for LongC were MESD, TNFSF14, SULT1A1, BST1 and MITD1. Principal component analysis (PCA) and Uniform Manifold Approximation and Projection (UMAP) analysis were also performed on the LongC cohort (Figure S1).

**Figure 4:**
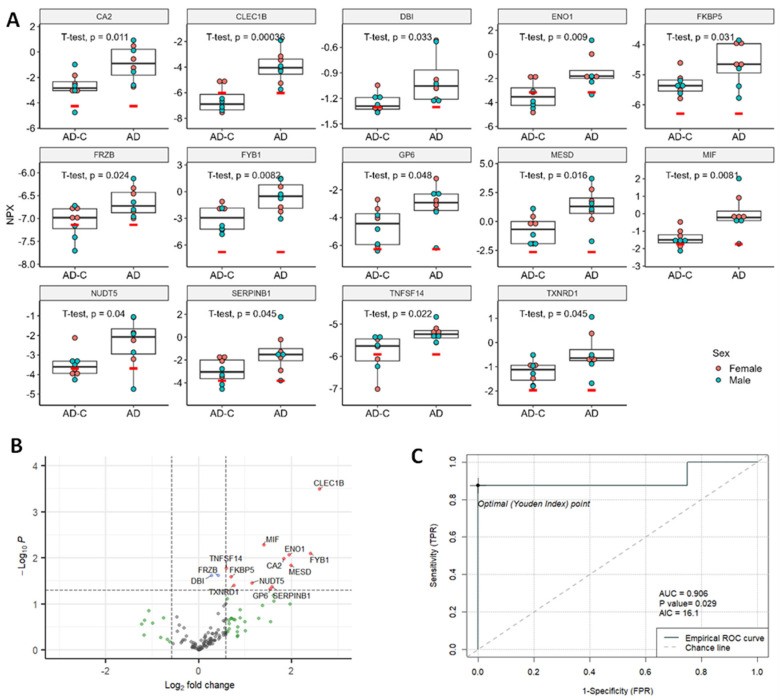
Significant DE proteins for AD cohort. (**A**) Dot plots of DE proteins. Unpaired, two-sided, unequal variance *t*-tests were used for comparisons of controls to AD. (**B**). Volcano plot. Vertical dashed lines indicate fold change of 1.5; horizontal line indicates *p* = 0.05. Red dots indicate proteins with fold changes >1.5 or <−1.5, and *p* values < 0.05; blue dots indicate proteins with fold changes of −1.5 to 1.5 and *p* values < 0.05; green dots indicate fold changes >1.5 or <−1.5, and *p* values ≥ 0.05; and gray dots indicate proteins with fold changes of −1.5 to 1.5, and *p* values ≥ 0.05. Red dots indicate significant DE proteins. Blue, green and gray dots indicate non-DE proteins. (**C**) ROC curve of the best predictor, MIF. NPX, normalized protein expression; AUC, area under the curve; AIC, Akaike information criterion; TPR, true positive rate, and FPR, false positive rate.

Fourteen DE proteins in the AD group were all significantly increased as seen in the dot plots (Figure 4A) and volcano plots (Figure 4B). The top five DE proteins used in ROC analysis were CLEC1B, MIF, TNFSF14, ENO1 and FYB1 (Table S2). The best predicting DE protein, MIF, showed an AUC of 0.906 (Figure 4C). PCA and UMAP analysis for the AD group can be found in Supplemental Figure S2.

**Figure 5:**
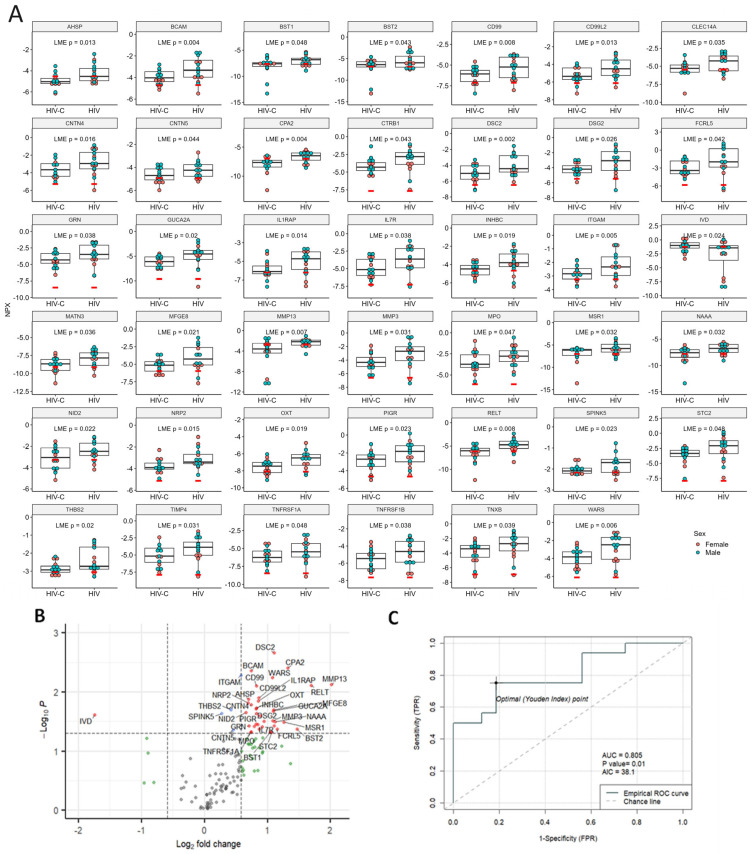
Significant DE proteins for HIV. (**A**) Dot plots of DE proteins. Linear mixed-effects models were used for comparisons of controls to HIV with age and sex as random effects and pair IDs as fixed effects. (**B**) Volcano plot. Vertical dashed lines indicate fold change of 1.5, horizontal line indicates *p* = 0.05. Red dots indicate proteins with fold changes >1.5 or <−1.5, and *p* values < 0.05; blue dots indicate proteins with fold changes of −1.5 to 1.5 and *p* values < 0.05; green dots indicate fold changes >1.5 or <−1.5, and *p* values ≥ 0.05; and gray dots indicate proteins with fold changes of −1.5 to 1.5, and *p* values ≥ 0.05. Red dots indicate significant DE proteins. Blue, green and gray dots indicate non-DE proteins. (**C**) ROC curve of the best predictor, CPA2, with an AUC of 0.805. NPX, normalized protein expression; AUC, area under the curve; AIC, Akaike information criterion; TPR, true positive rate, and FPR, false positive rate.

When we looked at the third cohort of nEVs from HIV+ cognitively impaired individuals compared to HIV+ cognitively unimpaired individuals, we found the greatest number of DE proteins with 40 proteins significantly increased and 1 protein, IVD, significantly decreased (Figure 5A,B). The top five DE proteins with the lowest AIC were CPA2, MMP13, RELT, AHSP and NRP2 (Table S2). The best predictor, CPA2, showed an AUC of 0.805 (Figure 5C). PCA and UMAP analysis for the HIV group are in Supplemental Figure S3.

**Figure 6:**
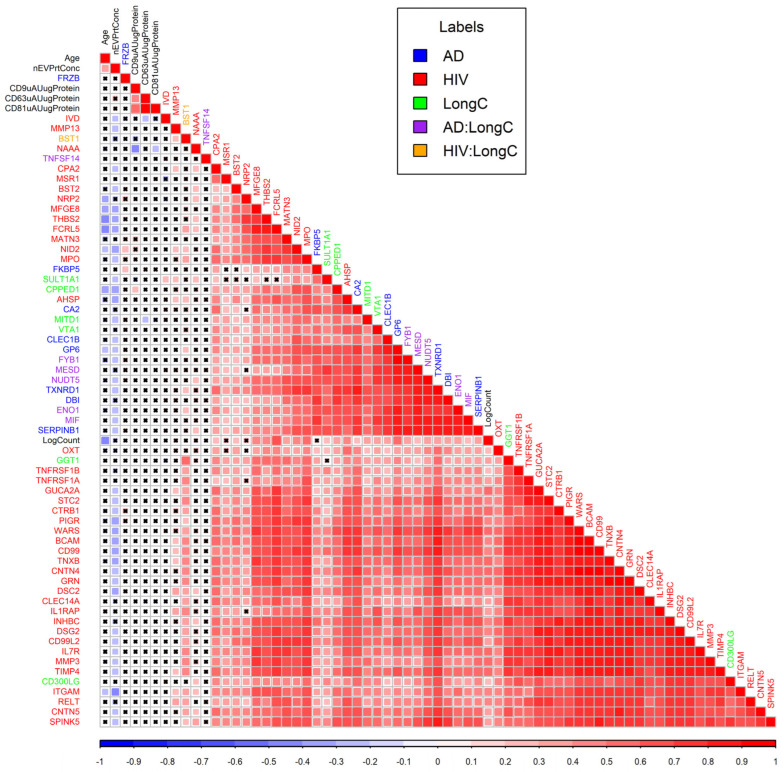
Correlation matrix plot for DE proteins of LongC, AD and HIV plus age, nEV concentrations, counts and tetraspanins. Colors of squares indicate Pearson correlation coefficients as shown in the scale bar under the matrix plot. Black crosses within squares indicate non-significant correlations of *p* values (Pearson’s correlations > 0.05). Colors of labels indicate groups as shown in the legend. Hierarchical clustering was used to arrange the targets in the plot. AD:LongC indicates targets were in both AD and LongC cohorts, similarly, HIV:LongC indicates targets were in both HIV and LongC cohorts.

The correlations between each pair of the significant DE proteins from all three groups were plotted in a correlation matrix (Figure 6). LongC and AD have clustered together which means higher similarities. Proteins from HIV mostly clustered with HIV proteins indicating lower correlations with AD or LongC.

**Figure 7:**
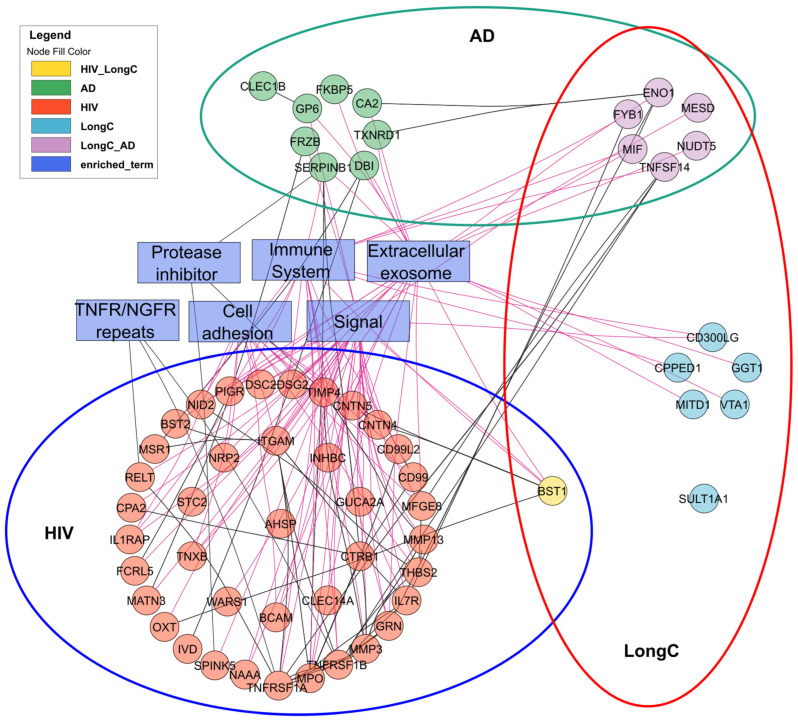
Interactions of significant DE proteins of LongC, AD and HIV cohorts and significantly associated functional terms analyzed with String DB and Cytoscape.

We evaluated related and divergent DE proteins between the three cohorts with significantly associated functional terms (Figure 7 and Table S3). In this panel of 384 neurological proteins, there were no common nEV DE proteins between HIV and AD. These distinct profiles may be useful in differentiating HIV cognitive impairment from AD. Conversely, there were six common proteins associated with LongC and AD. This adds to the common nEV DE proteins of Aβ and pTau181 previously reported [20]. It appears that these six DE proteins are mostly related to extracellular exosome- and immune-system-enriched functional terms (Table S4). Surprisingly, BST1 was the sole common DE protein in nEVs from LongC and HIV, displaying divergent changes between these infections.

## 3. Discussion

This pilot study was designed to discover neuronal proteins linking LongC, AD and HIV, as well as distinct proteins for each group. We also describe a new procedure to enrich scarce nEVs more efficiently in the plasma so that more exploratory studies can be performed. The EV-MAP technique has been efficiently used in isolating rare EVs associated with acute ischemic stroke [29]. We utilized the MISEV 2018 guidelines to characterize the nEVs using this hybrid technique and demonstrated that its advantage lies in the increased number of nEV numbers and protein with far less manual manipulation. Developing new techniques to rapidly isolate liquid markers from blood is a top priority in the field of personalized medicine such that samples can be easily analyzed longitudinally to time and treatments. New guidelines have been recently reported to standardize the isolation of extracellular vesicles from blood [27]. In addition, isolating enough EVs to further characterize any biomarkers identified by different -omics platforms is imperative. Ideally, a blood marker alone, without further isolations, would diagnose a condition, but this has been elusive for neurological diseases. One group has used the OLINK proximity multiplex platforms to compare plasma versus plasma EV proteins in AD [30]. The results showed that both plasma and EV proteins were able to distinguish AD from healthy controls, but EVs contained more proteins that were significantly different than controls. Also, the proteins of interest and significance between plasma and EVs were different. The EV proteins were thought to be more indicative of ongoing pathology. Additional interrogation of nEVs may give us an idea of neuronal health in real time. New neuronal specific markers on EVs, such as ATP1A3, which are less abundant in soluble form in blood, might curtail sequential separations and further facilitate rapid isolations [31].

We have previously analyzed nEV proteins from people with HIV and cognitive impairment [19]. The present goal of HIV clinical care is to maintain a sustained undetectable viral load with antiretroviral therapy (ART). However, with successful treatment, people living with HIV are aging and there is increasing interest in differentiating normal aging from HIV cognitive impairment and AD onset. With that in mind, biomarkers to separate these two conditions would be helpful. In this study of several hundred neurological proteins, there was no overlap of nEV proteins, making it easier to differentiate HIV and AD. In contrast, we previously reported that nEVs from people with LongC and neurological symptoms had proteins associated with AD markers [14,15,32,33], including increased Aβ and pTau181 [20]. The proteomic panel used in this study had new, unreported proteins and did not include Aβ and pTau181.

The one common protein between LongC and HIV was BST1 (bone marrow stromal cell antigen-1), also known as CD157. This protein is highly expressed in the embryonic brain where it regulates brain development [34]. In the adult brain, BST-1 expression is more restricted, being expressed in neural stem cells surrounding glial cells, functioning to regulate adult neurogenesis, migration and integration of newly formed neurons into existing circuitry [34]. BST1 is also expressed in dendritic cells and other cells of myelomonocytic lineage, thus playing a role in the innate immune response by controlling myeloid cell migration and diapedesis during inflammation [35]. Interestingly, BST1 is decreased in LongC (Figure 3A,B) and increased in HIV nEVs (Figure 5A,B). Gene knockout studies showed BST1^−/−^ mice displayed impaired social behavior, including increased anxiety, social avoidance/fear and depression-related behaviors that responded to antidepressant treatment [36,37]. These anxiety and depressive behaviors could also be linked to LongC.

Three of the best predictors for LongC proteins (MESD, TNFSF14 and BST1) have been linked to neuroinflammation and neurogenesis. MESD is a molecular chaperone for LRP5 and LRP6, and blocks Wnt/β-catenin-mediated cell proliferation and migration [38]. In the brain, blockage of Wnt signaling reduces neurogenesis in adult rat hippocampus [39]. TNFSF14, also known as the LIGHT protein, has been shown to play a role in dendritic cell-mediated immune response by costimulating human T cell proliferation [40]. Given these various reports, the findings in this study showing increased MESD, increased TNFSF14 and decreased BST1 may indicate the presence of neuroinflammation and diminished neurogenic capability in LongC individuals, which may affect the hippocampus region of the brain.

It is tempting to speculate that the difference in BST1 expression between LongC and HIV may indicate more subtle neurological differences between the two, such as severity of neuroinflammation or neurodegeneration, or location of brain injury. For instance, increased BST1 in HIV may indicate neuroinflammation and neurodegeneration affecting the cerebral cortex since studies have shown that BST1 expression is increased in astrocytes and microglia of Aβ-induced neurodegenerative mice [34] and manifestations of HIV cognitive dysfunction entail cortical involvement with impairments in memory/learning and executive function [41]. In contrast, decreased BST1 in LongC may entail decreased neurogenesis in the hippocampus, which controls emotions, such as anxiety and depression, in addition to memory and learning. Supporting this hypothesis are studies showing that stress and increased glucocorticoids can decrease hippocampal neurogenesis [42,43], and we recently found increased cortisol in LongC individuals [20]. Since the LongC subjects in this study did not undergo rigorous neurological clinical assessment, it is possible that a more stringent assessment may reveal neurological features that differ between HIV and LongC. Furthermore, the use of BST1 knockout mice to study HIV or SARS-CoV-2 infection may elucidate not only the neurological consequences of these viral infections but also validate BST1 as a therapeutic target for these diseases.

Macrophage migration inhibitory factor, MIF, is a pleiotropic protein that is important in regulating the inflammatory response. MIF is a cytokine involved in innate immunity and associated with tau accumulation, Aβ plaques and brain aging [44,45,46]. A significant increase in cerebrospinal fluid (CSF) MIF levels in AD plus a modulation in Aβ pathology in a murine transgenic model treated with a MIF inhibitor suggested that MIF may not only be a biomarker for AD but also a therapeutic target [46]. Of the six overlapping proteins between AD and LongC reported here, the best predictor of AD was MIF (AUC 9.06, Figure 4C). Previous studies show that MIF is elevated in a continuum in patients with mild cognitive impairment progressing to AD [47]. MIF is secreted from neurons and is thought to be a defense mechanism from the toxic effects of Aβ [47]. CSF MIF has been suggested as a biomarker to monitor neuroinflammation and progression of tau pathology in AD [48,49,50]. This has been supported in animal studies where knockout of the MIF protein attenuated tau phosphorylation, suggesting that this may be a therapeutic target [48]. MIF is also associated with major depressive disorder, and people with LongC have significant increases in depression and anxiety [51].

We previously analyzed nEVs from people with LongC and reported that many of the toxic proteins previously reported in nEVs from patients with AD, such as Aβ and pTau181 [14,15,32], were also in nEVs from people with LongC [19]. A recent study reported similarities between LongC and AD neuropathology [21]. Another study of omics comparisons between AD and survivors of COVID-19 found similar processes of neuroinflammation and brain microvascular injury [52].

This was a pilot study for both a new technique to isolate nEVs as well as differentiating pathways and markers for LongC, HIV and AD. The cohorts were small in number and the proteins of interest would need to be verified by other methods. Another limitation is that there may have been confounders that were not accounted for by the matching. These data add to the body of work showing similarities between AD and LongC at the EV and likely neuronal level [20]. There were significant differences in age between the LongC and AD cohorts; however, approximately half of the significant DE proteins from each group were in common. Longitudinal studies on LongC to monitor neurological symptoms and nEV cargo need to be conducted to determine if the proteins in common with AD are transient or permanent. Repositories for blood from people with complications of LongC need to be established that include those individuals who have recovered from LongC with time or treatment. Our study included some LongC participants up to 1 year post-infection. The proteins identified in this study would need to be verified with other techniques and combined with other proteins to establish a biomarker profile to be used in a longitudinal study. A recent study on LongC showed an increase in plasma IL-1β, IL-8, cortisol and nEV cargo of Aβ42, fibroblast growth factor 21 and high mobility group box B1 in people with neurological complications of LongC [20]. These analytes plus some identified in this report could be followed over time to monitor the progression or recovery of neurological impairment. Differentiating LongC from AD in aging individuals will be important as LongC may trigger or accelerate neuroinflammation, oxidative stress, tau phosphorylation and amyloid beta metabolism culminating in AD or an AD-like pathology. Studies suggesting links between infections and CNS disorders are also important and needed. Scientists have spent years looking for such connections in CNS diseases like multiple sclerosis, amyotrophic lateral sclerosis and AD. Does a virus or bacterial infection accelerate or exacerbate a chronic CNS disease? The implications are life-changing as one could only imagine a known antibiotic or antiviral as a cure for a devastating CNS disease. These -omics study comparisons and insights will be valuable for future research.

## 4. Materials and Methods

### 4.1. Plasma Collection

Plasma samples from individuals with LongC were recruited from the Veterans Affairs Health Care System (VAHCS), San Francisco and from advertising in social media from the San Francisco Bay area. Blood was drawn at the San Francisco VAHCS into EDTA tubes, aliquoted and frozen at −80 °C until use. All volunteers were at least 3 months post SARS-CoV-2 infection and had self-reported neurological symptoms of one or more of the following: memory difficulty, trouble concentrating, increased anxiety, or increased depression. Exclusion criteria involved any one of the following: past or present seizures, head trauma, loss of consciousness for more than 15 min, substance abuse or dependence (including alcohol) within 3 months of study participation, HIV and pregnancy. All participants signed a written informed consent approved by the University of California, San Francisco Institutional Review Board. Plasma samples from healthy HIV-negative controls, HIV+ individuals with normal cognition (NPN) and HIV+ individuals with mild cognitive impairment (MND) were obtained from the MACS/WIHS Combined Cohort Study (MWCCS). Characteristics and recruitment of participants in this study have been previously published [28]. Healthy control plasma used to characterize the hybrid EV-MAP procedure was purchased from Blood Centers of the Pacific (Vitalant, San Francisco, CA, USA). Plasma from AD patients and their age-matched controls was received from participants in NIH IRB-approved clinical studies run at the National Institute of Aging (NIA, Baltimore, MD, USA) before the COVID-19 pandemic. The AD patients had high-probability (early-stage) AD as defined in the NIA-AA criteria [53] based on clinical diagnosis and abnormal CSF levels of Aβ peptide 1–42 (Aβ42 < 192 pg/mL) and p181-Tau > 23pg/mL [54]; plasma samples used in this study were drawn during the baseline visit of a clinical trial of exenatide in early AD [55]. Corresponding controls were healthy, cognitively unimpaired age- and sex-matched individuals participating in NIA clinical studies. Samples received from outside sources were received in aliquots and frozen at −80 °C until use.

### 4.2. nEV Isolation

nEVs were isolated using a procedure previously described [32] with one major modification. Instead of using thromboplastin-D derived from rabbit brain tissue to clot the plasma and remove fibrils and coagulation proteins, we used thrombin to minimize contamination of the downstream isolated nEVs with unassociated brain proteins. Our modified procedure is as follows.

Thrombin (1.25 units, Sigma-Aldrich, Burlington, MA, USA, catalog # T4648) was added to 250 μL of plasma for 1 h at room temperature. The volume was then brought up to 500 μL with phosphate-buffered saline (PBS) containing 3× protease and phosphatase inhibitors (ThermoFisher Scientific, Rockford, IL, USA, catalog # 78446), centrifuged at 3000× *g* for 20 min, and the clarified plasma supernatant collected. Total EVs (tEV) were then polymer precipitated by adding 126 μL of ExoQuick^TM^ Exosome Precipitation Solution (Systems Biosciences, Palo Alto, CA, USA, catalog # EXOQ20A-1) to the clarified plasma in the presence of 3× protease and phosphatase inhibitors (ThermoFisher Scientific, Rockford, IL, USA, catalog # 78446) for 1 h at 4 °C, centrifuged at 100× *g* for 20 min at 4 °C, and the supernatant discarded. The precipitated tEV pellets were then resuspended in 350 μL of PBS containing 1× protease and phosphatase inhibitors and stored at −80 °C until loading onto EV-MAP microfluidic chip for immunoaffinity capture and purification of L1CAM+ EVs.

### 4.3. Microfluidic Affinity Purification of EVs (EV-MAP)

EV-MAP devices used in this study were purchased from BioFluidica, Inc. and fabricated in cyclic olefin polymer (COP, ZEONOR 1020/1060)) via injection molding (Stratec, Austria) from a mold insert made via UV-LiGA. The microfluidic network was comprised of 7 parallel selection beds (30 mm long and 4 mm wide) filled with diamond-shaped micropillars (10 × 10 µm) with the beds addressed using a single inlet and outlet channel (200 × 200 µm) arranged in the so-called z-configuration [25]. The device’s surfaces were activated with UV/O_3_ for the generation of surface COOH functionalities for the covalent attachment of mono-clonal antibody (mAb) with a photocleavable linker [56]. Before affinity isolation, all samples were centrifuged at 2000× *g* for 10 min to remove any large particles. The supernatant of samples was then loaded onto EV-MAP devices using the automated sample handling robot. Devices were blocked using 1% PVP/PBS before sample loading to eliminate non-specific binding and washed with 0.005% Tween 20 after EV isolation. Following affinity isolation, nEVs were released with visible light (405 nm) [56]. An automated sample-handling robot with custom software was used to operate microfluidics and streamline sample processing. The robot can process up to eight chips (i.e., samples) simultaneously. The robot pushes liquid using air displacement mode via pipettes that insert into the inlet and outlet ports on the chip. Fluid delivery to the microfluidic network is achieved by two pipetting channels operating simultaneously, one in “push” and one in “pull” mode. Such an approach eliminates cross-contamination and carryover errors as all components that come into direct contact with liquid samples, including pipette tips and fluidic chips, are disposable. The system is flexible in terms of volumes that can be delivered and flow rates for liquid delivery, which can be programmed into the operating protocol [25].

Purified nEV samples were stored at −80 °C until further downstream analyses.

### 4.4. Nanoparticle Tracking Analysis (NTA)

Light scattering technologies, such as NTA, are accepted methods of quantifying particle numbers and inferring their size. Thus, we performed NTA on the intact tEV and nEV samples using a ZetaView PMX 120 instrument (Particle Metrix GmbH, Inning am Ammersee, Germany) with a 488 nm laser-equipped chamber. Instrument calibration was performed using a known concentration of 100 nm polystyrene beads. Eleven positions were counted and analyzed using the ZetaView version 8.05.12 SP2 software. Each position was measured for 2 cycles using a sensitivity of 75 and a shutter value of 100. Triplicate readings were performed for each sample and the average was reported.

### 4.5. Transmission Electron Microscopy (TEM)

To provide structural and biomolecular composition information on the purified nEVs to complement the data obtained by NTA, TEM was performed on a subset of the intact nEVs isolated from patient plasma [57]. In brief, eluted nEVs were deposited onto Formvar carbon-coated electron microscopy nickel grids for 5 min. The excess fluid was blotted off with #1 filter paper and the grids were stained with saturated uranyl acetate solution (Ted Pella, Inc., Redding, CA, USA) for 5 s. Excess fluid was then blotted off again and the grids dried overnight. Visualization of EVs was performed using a Technai 10 transmission electron microscope (Field Electron and Ion Co., Hillsboro, OR, USA).

### 4.6. EV Lysate Preparation

EVs from the tEV and nEV fractions were lysed with M-PER^TM^ Mammalian Protein Extraction Reagent (ThermoFisher Scientific, Rockford, IL, USA, catalog # 78501) containing protease and phosphatase inhibitors, freeze–thawed twice and then stored at −80 °C until analysis by microBCA, ELISA and Olink Proteomics PEA (see below).

### 4.7. Protein Concentration Determination

Total protein concentrations of the plasma and lysates (plasma, tEV and nEV) were determined using the Pierce Micro BCA Protein Assay Reagent Kit (ThermoFisher Scientific, Waltham, MA, USA, catalog # 23235) per manufacturer’s instructions.

### 4.8. Enzyme-Linked Immunosorbent Assay (ELISA)

L1CAM, ApoB and ALB ELISAs were performed on unlysed plasma, tEVs and nEVs using the following commercially available ELISA kits: L1CAM (Invitrogen, ThermoFisher, Waltham, MA, USA, catalog # EH290RB); apolipoprotein B (ApoB, RayBiotech, Norcross, GA, USA, catalog # ELH-ApoB); and albumin (ALB, RayBiotech, Norcross, GA, USA, catalog # ELH-Albumin).

Alix and NeuN ELISA were performed on EV lysates. Purified EV fractions (tEVs and nEVs) were lysed with Mammalian Protein Extraction Reagent (M-PER^TM^, ThermoFisher Scientific, Waltham, MA,USA, catalog # 78501) containing protease and phosphatase inhibitors, freeze–thawed twice and then stored at −80 °C until analysis by ELISA. Alix (ALG-2-interacting protein X, also known as programmed cell death 6-interacting protein) protein levels were determined using an ELISA kit from Lifeome (Oceanside, CA, USA, catalog # CSB-EL017673HU) while NeuN (neuronal nuclei protein, also known as RNA binding protein fox-1 homolog [RBFOX3]) protein levels were determined using two different commercially available kits due to their availability at the time of assay. MyBioSource ELISA kit (San Diego, CA, USA, catalog # MBS7240544) was used to generate the NeuN data in Figure 1E, and an Abbexa LLC ELISA kit (Houston, TX, USA, catalog # abx541982) was used to generate the NeuN data in Figure 2C.

All ELISAs were performed according to manufacturer’s instructions and each sample was analyzed in duplicate. Protein concentrations were determined by absorbance using a Spectra Max M5 plate reader (Molecular Devices, San Jose, CA, USA) with Softmax^®^ Pro 7 version 7.1.2 software.

### 4.9. Meso Scale Discovery (MSD) Assay

Intact nEVs were quantified for tetraspanin protein levels using MSD assays for CD9, CD63, and CD81 (MSD, Rockville, MD, USA, custom kit catalog #K15228N-1 using antibody set catalog #s F215M-3-8, F2215L-3/-8 and F215N-3-8). All MSD assays were performed according to manufacturer’s instructions and each sample was tested in duplicate. All analyses were conducted using a QuickPlex SQ 120 instrument (MSD) and DISCOVERY WORKBENCH 4.0^®^ software.

### 4.10. Proximity Extension Assay (PEA)

All nEV lysate samples were adjusted to a concentration of 0.5 mg/mL for PEA analysis. Unique protein biomarkers (384 Neurology multiplex panel) were measured by PEA using the Olink Analysis Service at Olink laboratories (Olink Proteomics, Uppsala, Sweden). Data were normalized to standard plasma controls and expressed as normalized protein expression (NPX) values. NPX reflects relative protein concentration but does not absolutely quantify it. NPX is in a log2 scale and was used for comparisons between the samples.

### 4.11. Statistical Analysis

Pair-wise comparisons were performed using two-tailed *t*-tests for normally distributed values (Figure 1). For unpaired, non-normally distributed values, Mann–Whitney tests were used to compare group differences (Figure 2, Table 1).

For Olink PEA, the nEV lysate samples were diluted to 0.5 mg/mL and 1 µL sample was loaded for PEA analysis. The data was thus normalized to nEV protein concentrations. Low-quality NPX data were filtered out when returned from Olink. Only targets with 50% of the samples greater than the lower limit of detection (LOD) were included in the analyses. For accepted protein targets, data values below the LOD were included in Figure 3, Figure 4 and Figure 5 and analyses as recommended by the manufacturer since these do not commonly increase false positives and may reflect actual differences. Mean NPX were compared between groups with *t*-tests for LongC and AD. Linear mixed-effects model analysis was chosen for HIV samples which were rigorously paired for age, sex, neuropsychological status and HIV. Proteins with a *p*-value ≤ 0.05 were determined as significant. ROC curves were used to compare the efficacy of the targets and the lowest AIC was shown. Pearson’s correlation was used to determine correlations of DE proteins and ages, protein concentrations, nEV counts, and concentrations of tetraspanins. A correlation matrix plot was used to show potential relationships between the previous targets. Hierarchical clustering was used to arrange the targets in the correlation matrix plot. To explore the biological interactions of the proteins, the functional relationships of the DE proteins were analyzed with StringDB [58] and Cytoscape [59]. Six significant function terms were picked for relevance to the study. Statistical analyses were performed using R (version 4.3.2) [60].

## Figures and Tables

**Table 1:** Participant information.

		LongC (%)	LongC-C (%)	HIV (%)	HIV-C (%)	AD (%)	AD-C (%)
Sex	Female (*n* = 36)	4 (25)	8 (50)	8 (50)	8 (50)	4 (50)	4 (50)
	Male (*n* = 44)	12 (75)	8 (50)	8 (50)	8 (50)	4 (50)	4 (50)
Age in years, Mean ± SD		49.7 ± 10.8	51.3 ± 5.8	51.5 ± 6.1	52.1 ± 6.5	70.6 ± 5.4 *	70.3 ± 4.8

* *p* < 0.0001 AD vs. LongC, LongC-C, HIV and HIV-C (Mann-Whitney test: unpaired, non-parametric, two-tailed).

**Figure A1:**
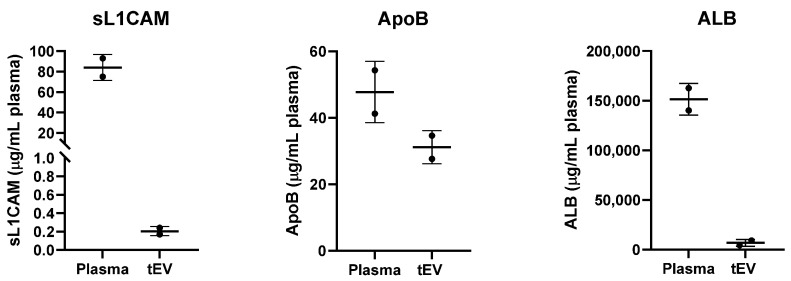
Plasma contaminants. Unlysed plasma and unlysed tEVs were analyzed by ELISA for the presence of soluble L1CAM, apolipoprotein B (ApoB) and albumin (ALB) proteins. Non-EV-associated plasma proteins were reduced by >99% for L1CAM (from 84 to 0.205 μg/mL plasma), 35% for ApoB (from 47.75 to 31.15 μg/mL plasma) and 96% for ALB (from 151,367 to 6,722 μg/mL plasma). Two different healthy control patient samples are shown here. This experiment was independently repeated two additional times with similar results.

**Figure A2:**
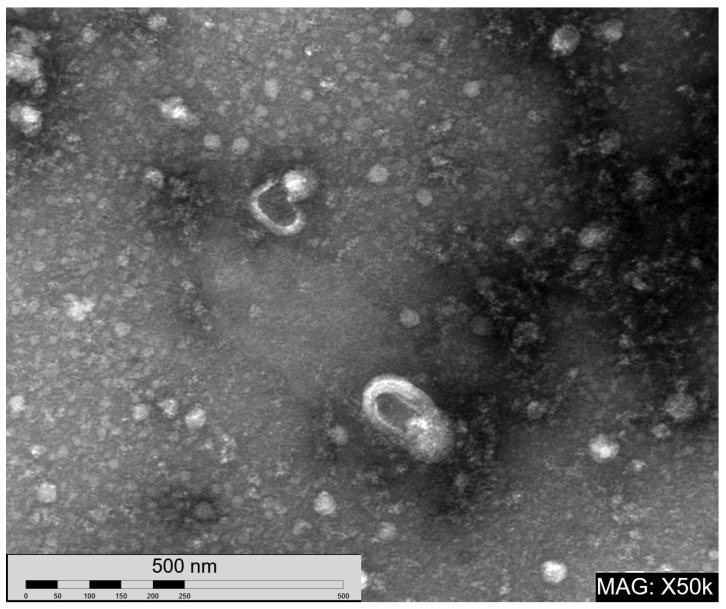
Transmission electron micrograph of nEVs. Magnification 50,000×.

## Data Availability

All raw data is available on request to corresponding author.

## Supplementary Materials

The following supporting information can be downloaded at: https://www.mdpi.com/article/10.3390/ijms25073830/s1.
