# Supplementary material for: Microfluidic Isolation of Neuronal-Enriched Extracellular Vesicles Shows Distinct and Common Neurological Proteins in Long COVID, HIV Infection and Alzheimer’s Disease
Source: Int J Mol Sci. 2024 Mar 29;25(7):3830. doi: 10.3390/ijms25073830 (PMC11011771; doi:10.3390/ijms25073830)
Supplement: Supplementary file 1 [file ijms-25-03830-s001.zip › ijms-2897081-supplementary.pdf]

International Journal of *Molecular Sciences*

MDPI

Supplementary Data

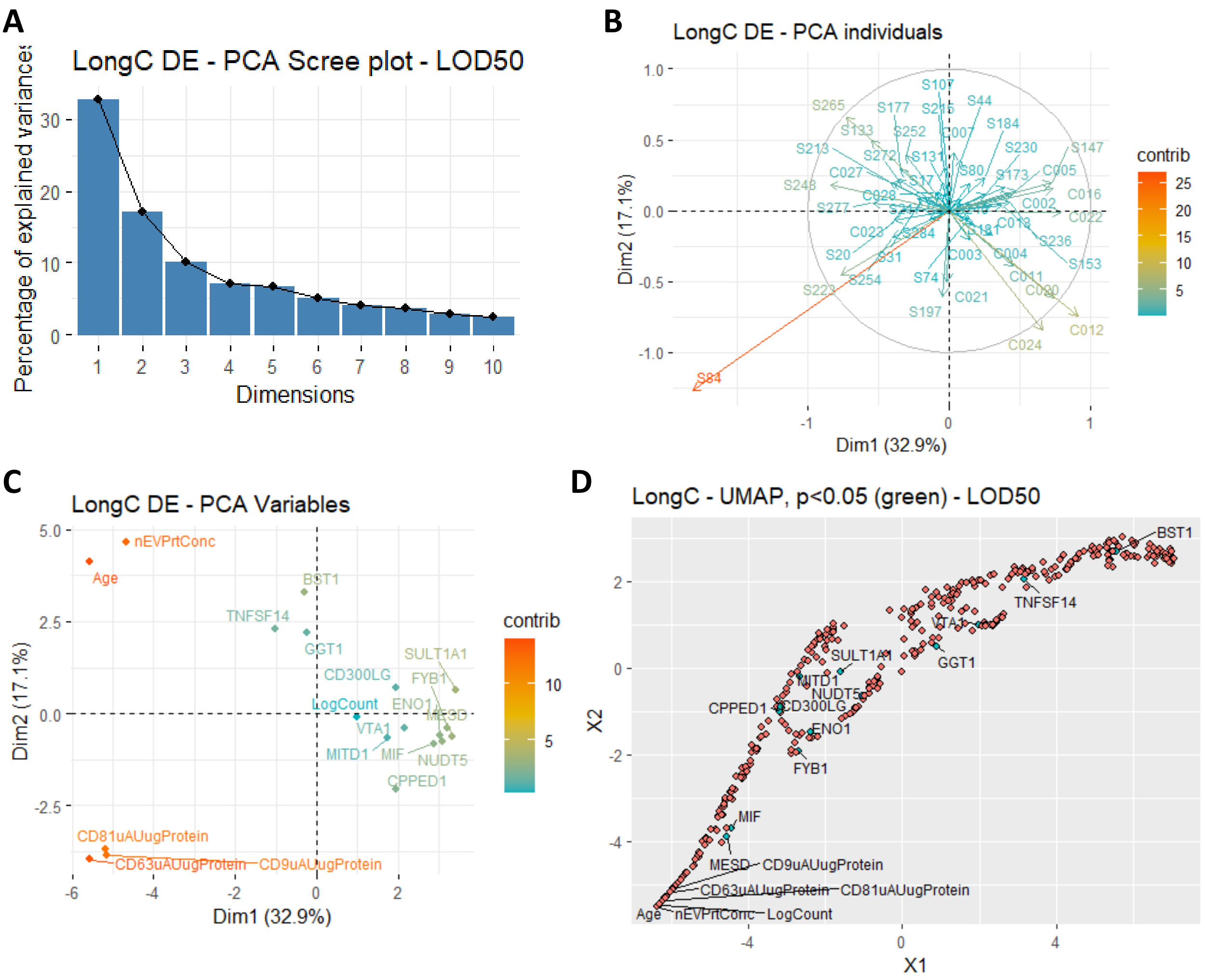

Figure S1. Principal component analysis (PCA) and Uniform Manifold Approximation and Projection (UMAP) analysis for LongC. All proteins with log10 nEV count (LogCount), nEV protein concentration by ELISA (nEVPrtConc), ELISAs of tetraspanins CD9, CD63, CD81 (uAUugProtein, micro arbitrary units per microgram of total nEV protein) and the age of the individual were included in the PCA and UMAP analyses. (**A**) Scree plot for the first 10 dimensions of the principal component analysis (PCA). (**B**) Individuals plot for PCA. (**C**) Variables plot for PCA. (**D**) UMAP plot. Green dots indicate DE proteins.

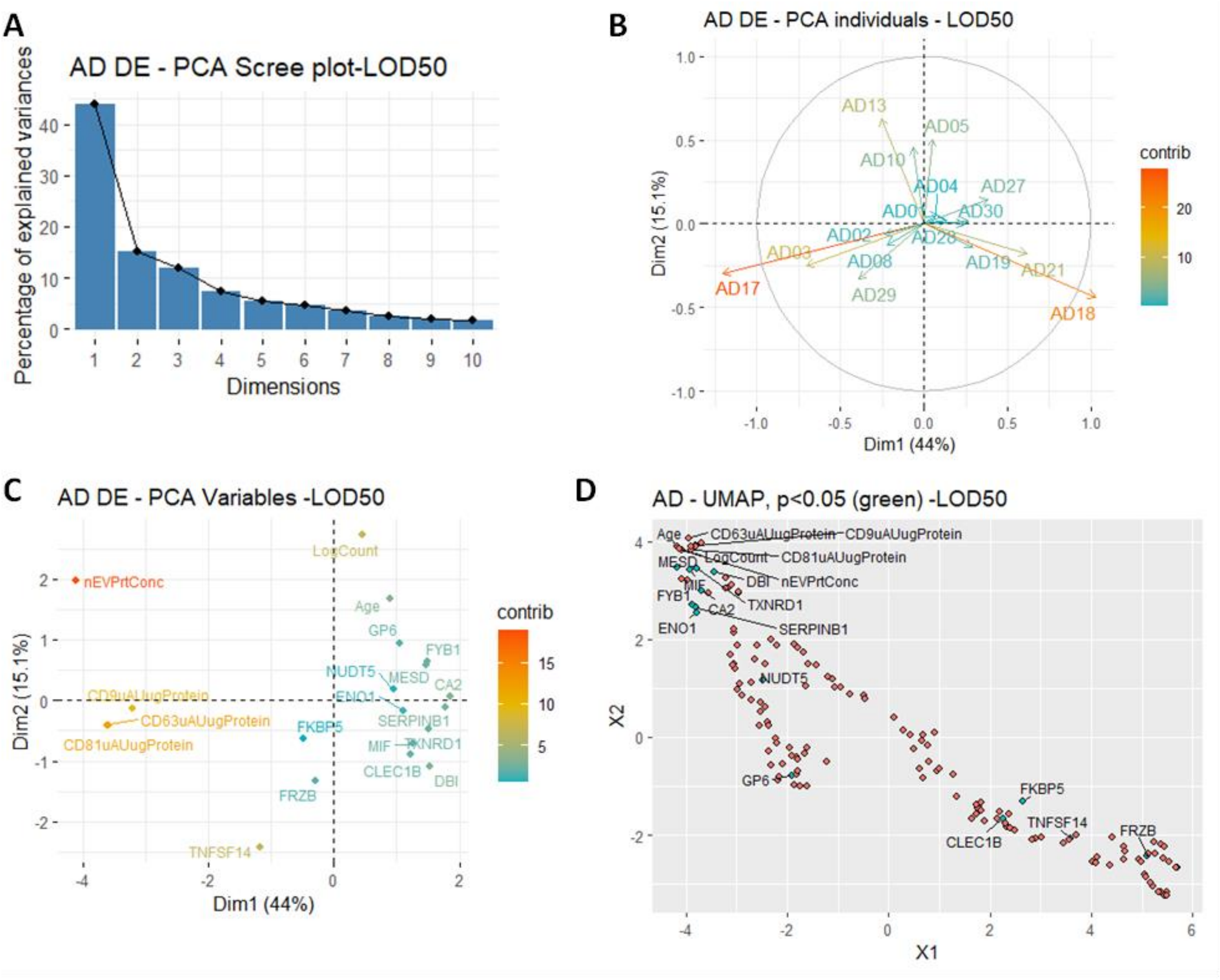

Figure S2. PCA and UMAP for AD. (**A**) Scree plot for the first 10 dimensions. (**B**) Individuals plot for PCA. (**C**) Variables plot for PCA. (**D**) UMAP plot for proteins and clinical variables and tetraspanins. Green dots indicate DE proteins.

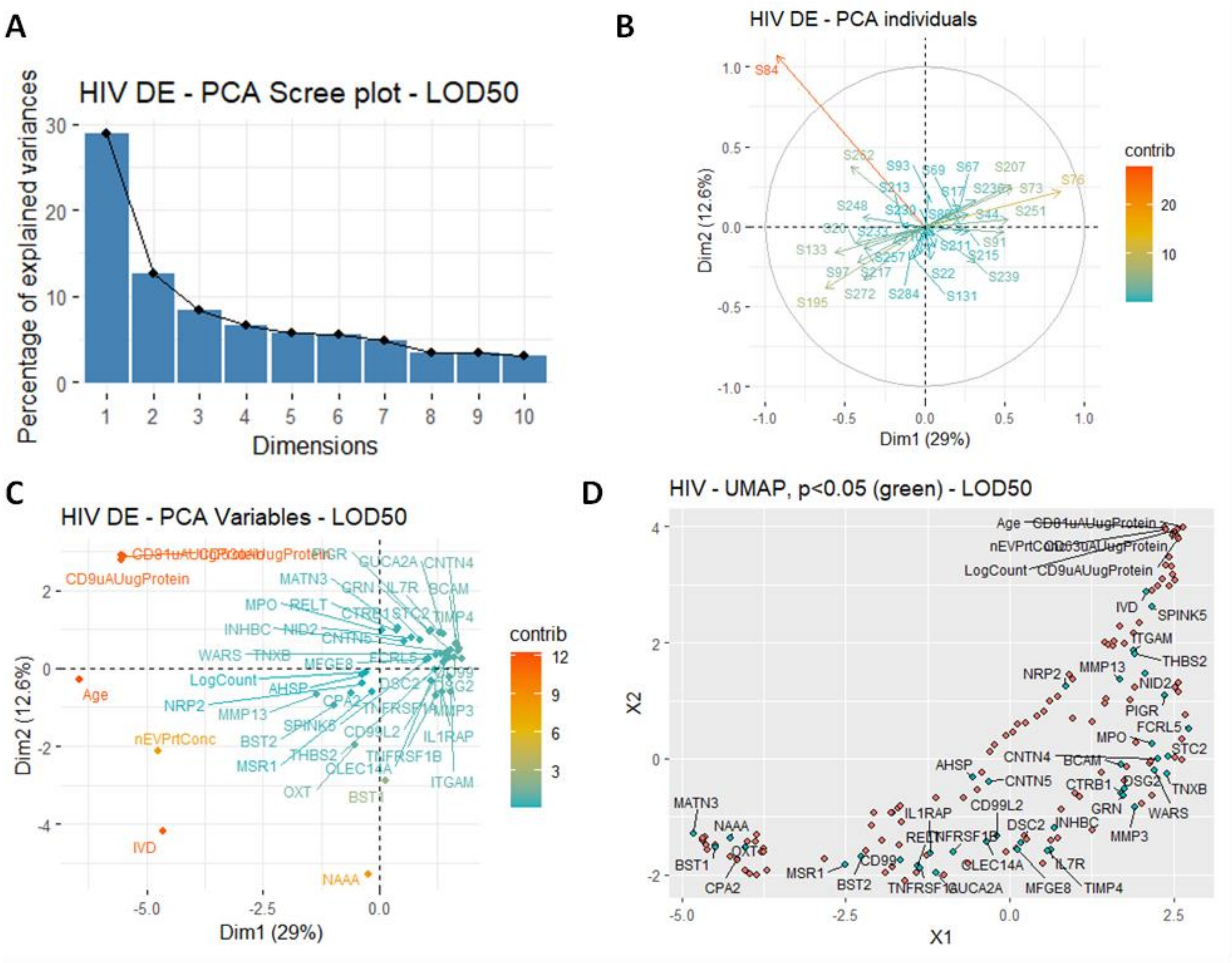

Figure S3. PCA and UMAP for HIV. (**A**) Scree plot for the first 10 dimensions. (**B**) Individuals plot for PCA. (**C**) Variables plot for PCA. (**D**) UMAP plot for proteins and clinical variables and tetraspanins. Green dots indicate DE proteins.

Table S1. DE proteins for LongC, AD and HIV cohorts.

Assay	LongC-C	LongC	p.LongC	FC.LongC	AD-C	AD	p.AD	FC.AD	HIV-C	HIV	p.HIV	FC.HIV
AHSP	-4.07 ± 1.00	-3.62 ± 1.37			-4.45 ± 0.44	-4.34 ± 1.16			-4.97 ± 0.64	-4.26 ± 0.86	0.013	1.63
BCAM	-3.44 ± 0.91	-3.31 ± 1.14			-3.51 ± 0.84	-3.30 ± 1.19			-3.99 ± 0.66	-3.25 ± 1.14	0.004	1.67
BST1	-6.94 ± 0.74	-8.08 ± 1.69	0.020	0.45	-6.89 ± 1.11	-7.35 ± 0.98			-8.06 ± 1.86	-7.01 ± 0.97	0.048	2.07
BST2	-5.53 ± 1.23	-5.85 ± 1.34			-6.08 ± 1.04	-7.24 ± 2.38			-7.01 ± 2.32	-5.54 ± 1.69	0.043	2.78
CA2	-2.84 ± 1.80	-1.60 ± 1.79			-2.73 ± 1.09	-0.90 ± 1.37	0.010	3.56	-3.26 ± 1.25	-2.73 ± 1.56		
CD300LG	-2.83 ± 0.40	-2.47 ± 0.47	0.026	1.28	-2.50 ± 0.54	-2.37 ± 0.49			-2.67 ± 0.37	-2.44 ± 0.67		
CD99	-5.53 ± 1.15	-5.29 ± 1.51			-5.76 ± 0.98	-5.20 ± 1.23			-6.21 ± 0.99	-5.39 ± 1.36	0.008	1.77
CD99L2	-4.53 ± 1.14	-4.41 ± 1.44			-4.74 ± 0.61	-4.47 ± 1.28			-5.27 ± 0.94	-4.40 ± 1.16	0.013	1.82
CLEC14A	-5.13 ± 1.22	-5.29 ± 1.16			-4.71 ± 0.99	-5.05 ± 1.46			-5.40 ± 1.13	-4.58 ± 1.25	0.035	1.76
CLEC1B	-3.99 ± 1.80	-3.25 ± 2.02			-6.59 ± 0.98	-3.99 ± 1.20	0.000	6.07	-5.77 ± 2.30	-6.13 ± 2.37		
CNTN4	-2.94 ± 1.12	-2.80 ± 1.63			-3.02 ± 1.24	-2.96 ± 1.44			-3.57 ± 0.95	-2.82 ± 1.38	0.016	1.68
CNTN5	-4.20 ± 0.56	-4.17 ± 1.14			-4.47 ± 0.57	-4.27 ± 0.96			-4.67 ± 0.64	-4.22 ± 0.70	0.044	1.37
CPA2	-6.78 ± 1.11	-6.92 ± 1.46			-7.65 ± 2.37	-6.81 ± 1.36			-8.04 ± 1.44	-6.71 ± 0.94	0.004	2.50
CPPED1	-2.67 ± 0.20	-2.43 ± 0.41	0.042	1.18	-2.80 ± 0.20	-2.74 ± 0.25			-2.75 ± 0.17	-2.71 ± 0.19		
CTRB1	-3.77 ± 1.17	-3.06 ± 1.59			-3.98 ± 0.67	-3.73 ± 1.33			-4.16 ± 1.06	-3.29 ± 1.71	0.043	1.83
DBI	-1.15 ± 0.24	-0.87 ± 0.53			-1.25 ± 0.11	-0.97 ± 0.29	0.024	1.21	-1.33 ± 0.15	-1.29 ± 0.18		
DSC2	-4.43 ± 1.13	-3.98 ± 1.14			-4.33 ± 1.36	-4.03 ± 1.99			-5.08 ± 1.12	-3.97 ± 1.30	0.002	2.15
DSG2	-3.54 ± 0.99	-3.31 ± 1.38			-3.69 ± 1.02	-3.48 ± 1.46			-4.20 ± 0.79	-3.32 ± 1.71	0.026	1.84
ENO1	-2.19 ± 1.25	-1.06 ± 1.84	0.050	2.20	-3.42 ± 1.13	-1.47 ± 1.41	0.009	3.87	-3.16 ± 1.30	-2.91 ± 1.15		
FCRL5	-1.95 ± 1.67	-2.12 ± 1.85			-4.33 ± 0.96	-4.20 ± 1.04			-3.05 ± 1.32	-1.90 ± 2.36	0.042	2.23
FKBP5	-5.26 ± 0.65	-4.80 ± 1.16			-5.32 ± 0.36	-4.63 ± 0.70	0.026	1.62	-5.47 ± 0.68	-5.32 ± 0.63		
FRZB	-6.70 ± 0.69	-6.82 ± 0.28			-7.06 ± 0.35	-6.65 ± 0.31	0.024	1.34	-7.01 ± 0.48	-6.83 ± 0.37		
FYB1	-0.89 ± 2.43	1.24 ± 2.34	0.017	4.36	-2.98 ± 1.42	-0.57 ± 1.68	0.008	5.32	-2.51 ± 2.29	-2.46 ± 2.13		
GGT1	-4.75 ± 0.81	-5.50 ± 1.15	0.042	0.60	-4.81 ± 0.61	-4.79 ± 1.20			-5.20 ± 1.30	-4.65 ± 1.51		
GP6	-2.92 ± 2.09	-1.60 ± 2.15			-4.64 ± 1.37	-3.11 ± 1.46	0.048	2.89	-4.49 ± 1.71	-4.43 ± 1.89		
GRN	-3.86 ± 1.10	-3.79 ± 1.54			-3.90 ± 0.96	-3.88 ± 1.23			-4.24 ± 1.11	-3.54 ± 1.62	0.038	1.63
GUCA2A	-5.39 ± 1.25	-5.59 ± 2.02			-5.51 ± 1.27	-5.43 ± 1.49			-6.11 ± 1.08	-5.01 ± 2.32	0.020	2.14
IL1RAP	-4.91 ± 0.93	-5.10 ± 1.41			-4.70 ± 1.08	-4.12 ± 1.84			-5.89 ± 0.97	-5.03 ± 1.26	0.014	1.81
IL7R	-3.88 ± 1.57	-3.98 ± 2.18			-4.27 ± 1.14	-3.94 ± 1.42			-4.90 ± 1.42	-3.79 ± 1.93	0.038	2.16
INHBC	-3.98 ± 0.89	-3.50 ± 1.60			-3.94 ± 0.82	-3.91 ± 1.31			-4.59 ± 0.77	-3.77 ± 1.34	0.019	1.77
ITGAM	-2.41 ± 0.79	-2.31 ± 0.78			-3.04 ± 1.00	-2.91 ± 1.24			-2.80 ± 0.59	-2.22 ± 0.95	0.005	1.50
IVD	-2.85 ± 3.42	-1.39 ± 1.95			-1.44 ± 3.01	-2.06 ± 2.44			-1.12 ± 1.00	-2.86 ± 3.06	0.024	0.30
MATN3	-7.91 ± 0.96	-7.94 ± 1.49			-8.72 ± 0.60	-8.14 ± 0.71			-8.80 ± 1.06	-7.95 ± 1.14	0.036	1.80
MESD	-0.34 ± 1.53	1.42 ± 1.71	0.004	3.40	-0.72 ± 1.18	1.27 ± 1.64	0.015	3.97	-1.17 ± 1.01	-1.05 ± 1.19		
MFGE8	-5.06 ± 2.89	-4.40 ± 1.89			-6.29 ± 1.37	-6.28 ± 1.24			-5.24 ± 0.94	-4.15 ± 1.84	0.021	2.13
MIF	-0.91 ± 1.06	0.16 ± 1.63	0.036	2.10	-1.42 ± 0.51	-0.021 ± 1.09	0.005	2.64	-1.62 ± 0.93	-1.58 ± 0.86		
MITD1	-3.30 ± 0.63	-2.65 ± 0.85	0.020	1.57	-3.26 ± 0.48	-3.26 ± 0.84			-3.19 ± 0.53	-3.33 ± 0.53		
MMP13	-2.98 ± 1.08	-2.34 ± 1.16			-2.30 ± 0.50	-2.71 ± 0.94			-4.40 ± 2.98	-2.38 ± 0.81	0.007	4.06
MMP3	-3.60 ± 1.41	-3.91 ± 1.89			-4.35 ± 1.40	-4.09 ± 1.63			-4.27 ± 1.21	-3.19 ± 2.04	0.031	2.12
MPO	-2.56 ± 1.06	-2.71 ± 1.35			-3.78 ± 0.69	-3.07 ± 1.36			-3.50 ± 1.20	-2.76 ± 1.17	0.047	1.67
MSR1	-6.62 ± 2.11	-6.47 ± 1.99			-6.53 ± 2.78	-7.07 ± 3.34			-7.01 ± 1.97	-5.75 ± 1.35	0.032	2.40
NAAA	-6.92 ± 1.03	-6.62 ± 0.75			-7.95 ± 1.67	-7.17 ± 0.45			-8.02 ± 1.72	-6.90 ± 1.02	0.032	2.18
NID2	-2.00 ± 1.56	-1.57 ± 1.53			-3.05 ± 0.61	-2.74 ± 0.93			-3.11 ± 1.09	-2.45 ± 0.87	0.022	1.58
NRP2	-3.51 ± 0.86	-3.22 ± 0.81			-4.11 ± 0.56	-3.85 ± 0.47			-3.79 ± 0.61	-3.09 ± 0.89	0.015	1.62
NUDT5	-3.03 ± 1.37	-1.60 ± 1.85	0.018	2.70	-3.52 ± 0.65	-2.37 ± 1.22	0.035	2.21	-3.51 ± 1.33	-3.23 ± 1.13		
OXT	-6.61 ± 1.00	-6.56 ± 1.49			-6.63 ± 0.63	-7.09 ± 0.72			-7.57 ± 0.85	-6.74 ± 1.03	0.019	1.78
PIGR	-2.29 ± 1.17	-1.74 ± 1.66			-2.59 ± 0.93	-2.36 ± 1.17			-2.94 ± 1.13	-2.11 ± 1.30	0.023	1.77
RELT	-5.75 ± 1.00	-5.19 ± 1.84			-5.64 ± 0.88	-5.73 ± 1.50			-6.58 ± 1.99	-4.89 ± 1.49	0.008	3.24
SERPINB1	-2.08 ± 1.34	-1.23 ± 1.72			-2.98 ± 1.08	-1.41 ± 1.68	0.042	2.98	-2.95 ± 1.34	-2.90 ± 1.16		

SPINK5	-1.84 ± 0.36	-1.56 ± 0.64			-1.85 ± 0.47	-1.73 ± 0.65			-2.04 ± 0.22	-1.76 ± 0.47	0.023	1.21
STC2	-2.78 ± 1.36	-2.82 ± 2.57			-2.84 ± 1.11	-2.97 ± 1.81			-3.61 ± 1.38	-2.55 ± 2.11	0.048	2.09
SULT1A1	-3.87 ± 2.65	-1.66 ± 1.95	0.012	4.64	-3.81 ± 2.81	-1.84 ± 1.46			-4.05 ± 2.35	-3.25 ± 0.94		
THBS2	-2.42 ± 0.73	-2.52 ± 0.62			-3.15 ± 0.11	-3.16 ± 0.18			-2.84 ± 0.33	-2.42 ± 0.72	0.020	1.34
TIMP4	-4.00 ± 1.44	-4.18 ± 1.82			-4.59 ± 1.41	-4.55 ± 1.77			-5.11 ± 1.48	-4.17 ± 1.84	0.031	1.92
TNFRSF1A	-5.67 ± 1.05	-6.30 ± 2.76			-5.19 ± 1.36	-4.65 ± 1.55			-6.13 ± 1.13	-5.39 ± 1.58	0.048	1.67
TNFRSF1B	-5.63 ± 2.06	-5.68 ± 1.62			-5.21 ± 1.00	-4.64 ± 1.64			-5.56 ± 1.13	-4.77 ± 1.46	0.038	1.73
TNFSF14	-6.18 ± 1.85	-5.14 ± 0.52	0.039	2.06	-5.88 ± 0.56	-5.28 ± 0.26	0.017	1.51	-5.74 ± 0.47	-5.43 ± 0.53		
TNXB	-2.96 ± 1.36	-2.93 ± 1.88			-3.30 ± 1.56	-3.66 ± 1.50			-3.88 ± 1.48	-2.96 ± 1.73	0.039	1.89
TXNRD1	-0.89 ± 0.72	-0.24 ± 1.27			-1.21 ± 0.46	-0.45 ± 0.83	0.040	1.69	-1.35 ± 0.50	-1.05 ± 0.77		
VTA1	-4.93 ± 3.09	-2.97 ± 2.15	0.046	3.89	-4.63 ± 1.35	-3.79 ± 3.26			-5.17 ± 0.93	-4.84 ± 1.52		
WARS	-3.06 ± 1.45	-2.56 ± 1.74			-3.52 ± 1.18	-3.07 ± 1.58			-3.90 ± 1.00	-2.82 ± 1.53	0.006	2.12

The table showed mean ± S.D of NPX. T tests were used to determine group differences between impaired and NPN for LongC and AD. Mixed-effect linear model was used for HIV to take care of the paired data. FC stands for fold changes of impaired group and NPN controls.

Table S2. Top 5 DE proteins of the ROC analysis with cognitive impairment for each group.

LongC			
**Target**	**AUC**	**P**	**AIC**
MESD	0.793	0.013	39.77
TNFSF14	0.766	0.030	40.35
SULT1A1	0.730	0.031	40.84
BST1	0.797	0.032	40.92
MITD1	0.742	0.038	42.29
AD			
**Target**	**AUC**	**P**	**AIC**
CLEC1B	0.938	0.053	12.41
MIF	0.906	0.029	16.15
TNFSF14	0.906	0.090	16.98
ENO1	0.891	0.048	17.65
FYB1	0.859	0.048	18.28
HIV			
**Target**	**AUC**	**P**	**AIC**
CPA2	0.805	0.010	38.09
MMP13	0.750	0.052	39.98
RELT	0.781	0.025	40.55
AHSP	0.734	0.031	41.56
NRP2	0.777	0.035	41.73

AUC, area under the curve; AIC, Akaike information criterion. Logistic regression was used to determine the predictive value of the target to cognitive impairment.

Table S3. Functional analysis of the DE proteins from the 3 groups using Gene Set Enrichment Analysis.

		LongC				AD				LongC			
**ID**	**Description**	**size**	**P value**	**NES**	**Symbol**	**size**	**P value**	**NES**	**Symbol**	**size**	**P value**	**NES**	**Symbol**
**GO:0008104**	protein localization	3	0.04	1.587	FYB1,VTA1,MESD	2	0.053	1.479	FYB1,MESD	4	0.946	0.583	MSR1
**GO:0051641**	cellular localization	3	0.04	1.587	FYB1,VTA1,MESD	2	0.053	1.479	FYB1,MESD	5	0.789	-0.808	TNFRSF1A,GRN,ITGAM
**GO:0051668**	localization within membrane	2	0.175	1.292	FYB1,MESD	2	0.053	1.479	FYB1,MESD	3	0.225	-1.253	TNFRSF1A,ITGAM
**GO:0070727**	cellular macromolecule localization	3	0.04	1.587	FYB1,VTA1,MESD	2	0.053	1.479	FYB1,MESD	4	0.946	0.583	MSR1
**GO:0072657**	protein localization to membrane	2	0.175	1.292	FYB1,MESD	2	0.053	1.479	FYB1,MESD	3	0.225	-1.253	TNFRSF1A,ITGAM
**GO:0048468**	cell development					2	0.081	1.391	CLEC1B	10	0.012	-1.857	TNFRSF1B,CNTN4,TNFRSF1A,GRN,AHSP,NRP2,CNTN5,SPINK5
**GO:0045595**	regulation of cell differentiation					2	0.075	-1.567	FRZB	8	0.813	-0.731	TNFRSF1B,CNTN4,TNFRSF1A,GRN,SPINK5
**GO:0045597**	positive regulation of cell differentiation					2	0.075	-1.567	FRZB	4	0.578	0.95	MSR1,IL7R,TNXB
**GO:0050793**	regulation of developmental process					2	0.075	-1.567	FRZB	13	0.992	0.46	MSR1,IL7R,WARS1,STC2,TNXB,IL1RAP,OXT,TNFRSF1B,CNTN4,TNFRSF1A,GRN
**GO:0051094**	positive regulation of developmental process					2	0.075	-1.567	FRZB	9	0.993	0.459	MSR1,IL7R,TNXB,IL1RAP,OXT,TNFRSF1B,TNFRSF1A,GRN
**GO:0009887**	animal organ morphogenesis									3	0.007	1.69	MMP13,RELT
**GO:0010605**	negative regulation of macromolecule metabolic process	3	1	0	MIF,TNFSF14	5	0.869	-0.759	DBI	6	0.038	1.591	BST2,MSR1,WARS1,STC2,TIMP4
**GO:0030029**	actin filament-based process									5	0.053	1.518	BST2,DSC2,BST1,TNXB,DSG2
**GO:0040007**	growth					2	0.661	0.926	ENO1	4	0.092	1.419	MMP13,BST2
**GO:0010629**	negative regulation of gene expression					2	0.794	0.807	SERPINB1,MIF	4	0.099	1.411	BST2,MSR1,STC2
**GO:0050728**	negative regulation of inflammatory response									3	0.093	-1.47	TNFRSF1A,GRN
**GO:0070997**	neuron death									3	0.093	-1.47	GRN,ITGAM
**GO:0097190**	apoptotic signaling pathway	2	0.773	0.861	ENO1,MIF	2	0.794	0.807	ENO1,MIF	3	0.093	-1.47	TNFRSF1A,ITGAM
**GO:1901214**	regulation of neuron death									3	0.093	-1.47	GRN,ITGAM
**GO:0030182**	neuron differentiation									6	0.093	-1.496	CNTN4,GRN,NRP2,CNTN5,SPINK5
**GO:0048699**	generation of neurons									6	0.093	-1.496	CNTN4,GRN,NRP2,CNTN5,SPINK5
**GO:0000904**	cell morphogenesis involved in differentiation									3	0.073	-1.525	NRP2,CNTN5
**GO:0007409**	axonogenesis									3	0.073	-1.525	NRP2,CNTN5
**GO:0007411**	axon guidance									3	0.073	-1.525	NRP2,CNTN5
**GO:0032989**	cellular component morphogenesis									3	0.073	-1.525	NRP2,CNTN5
**GO:0032990**	cell part morphogenesis									3	0.073	-1.525	NRP2,CNTN5
**GO:0048667**	cell morphogenesis involved in neuron differentiation									3	0.073	-1.525	NRP2,CNTN5

**GO:0048812**	neuron projection morphogenesis									3	0.073	-1.525	NRP2,CNTN5
**GO:0048858**	cell projection morphogenesis									3	0.073	-1.525	NRP2,CNTN5
**GO:0097485**	neuron projection guidance									3	0.073	-1.525	NRP2,CNTN5
**GO:0120039**	plasma membrane bounded cell projection morphogenesis									3	0.073	-1.525	NRP2,CNTN5
**GO:0034330**	cell junction organization									7	0.078	-1.545	IL1RAP,OXT,NRP2,ITGAM,CNTN5,THBS2
**GO:0050808**	synapse organization									6	0.063	-1.616	OXT,NRP2,ITGAM,CNTN5,THBS2
**GO:0031348**	negative regulation of defense response									4	0.03	-1.737	TNFRSF1A,GRN,SPINK5
**GO:0032102**	negative regulation of response to external stimulus									4	0.03	-1.737	TNFRSF1A,GRN,SPINK5
**GO:0043065**	positive regulation of apoptotic process									4	0.03	-1.737	TNFRSF1A,GRN,ITGAM
**GO:0043068**	positive regulation of programmed cell death									4	0.03	-1.737	TNFRSF1A,GRN,ITGAM
**GO:0022008**	neurogenesis									7	0.031	-1.743	TNFRSF1B,CNTN4,GRN,NRP2,CNTN5,SPINK5
**GO:0030154**	cell differentiation					3	0.218	1.256	CLEC1B	12	0.026	-1.757	TNFRSF1B,CNTN4,TNFRSF1A,GRN,AHSP,NRP2,ITGAM,CNTN5,SPINK5
**GO:0048869**	cellular developmental process					3	0.218	1.256	CLEC1B	12	0.026	-1.757	TNFRSF1B,CNTN4,TNFRSF1A,GRN,AHSP,NRP2,ITGAM,CNTN5,SPINK5
**GO:0007420**	brain development									4	0.02	-1.801	NRP2,ITGAM,CNTN5
**GO:0060322**	head development									4	0.02	-1.801	NRP2,ITGAM,CNTN5
**GO:0061564**	axon development									4	0.02	-1.801	GRN,NRP2,CNTN5
**GO:0007417**	central nervous system development									8	0.009	-1.969	TNFRSF1B,CNTN4,GRN,NRP2,ITGAM,CNTN5,SPINK5
**GO:0007399**	nervous system development									12	0.008	-1.97	TNXB,IL1RAP,OXT,TNFRSF1B,CNTN4,GRN,NRP2,ITGAM,CNTN5,THBS2,SPINK5
**GO:0031347**	regulation of defense response	2	0.035	-1.65	BST1					6	0.594	-0.889	TNFRSF1B,TNFRSF1A,GRN,SPINK5
**GO:0050727**	regulation of inflammatory response	2	0.035	-1.65	BST1					5	0.784	-0.81	BST1,TNFRSF1B,TNFRSF1A,GRN

Gene set enrichment analysis was performed using R (version 4.3.2) and clusterProfiler package (version 4.8.3). NES indicates normalized enrichment score.

Table S4. Functional annotations of DE proteins.

category	term ID	term description	observed gene count	background gene count	strength	false discovery rate
GO Component	GO:0070062	Extracellular exosome	28	2096	0.62	6.29E-09
UniProt Keywords	KW-0732	Signal	40	3277	0.58	4.42E-14
UniProt Keywords	KW-0646	Protease inhibitor	4	117	1.03	0.0338
GO Process	GO:0007155	Cell adhesion	14	965	0.66	0.0295
Reactome	HSA-168256	Immune System	18	1979	0.45	0.0385
SMART	SM00208	Tumor necrosis factor receptor / nerve growth factor receptor repeats.	3	24	1.59	0.0351

Strength indicates Log10(observed / expected). This measure describes how large the enrichment effect is. It’s the ratio between i) the number of proteins in your network that are annotated with a term and ii) the number of proteins that we expect to be annotated with this term in a random network of the same size. False Discovery Rate describes how significant the enrichment is. Shown are *p* values corrected for multiple testing within each category using the Benjamini–Hochberg procedure. Explanations are adapted from StringDB.
